# Testing the Effectiveness of a Community-Based Peer Support Intervention to Mitigate Social Isolation and Stigma of Adolescent Motherhood in Zimbabwe

**DOI:** 10.1007/s10995-023-03821-2

**Published:** 2023-11-13

**Authors:** Chiwoneso B. Tinago, Edward A. Frongillo, Andrea M. Warren, Vivian Chitiyo, Tiara N. Jackson, Ashley K. Cifarelli, Shannon Fyalkowski, Victoria Pauline

**Affiliations:** 1 Department of Public Health Sciences, West Chester University of Pennsylvania, 155 University Avenue, West Chester, PA 19383 USA; 2 Department of Health Promotion, Education, and Behavior, Arnold School of Public Health, University of South Carolina, 915 Greene Street, Columbia, SC 29208 USA; 3 The Organization for Public Health Interventions and Development (OPHID), 20 Cork Road, Belgravia, Harare, Zimbabwe; 4 NORC at the University of Chicago, 4350 East-West Highway, 8th Floor, Bethesda, MD USA

**Keywords:** Adolescent mothers, Peer support, Mental health, WhatsApp Messenger, Social isolation and stigma

## Abstract

**Objectives:**

Social isolation and stigma contribute to poor mental health outcomes. Adolescent mothers in Zimbabwe often experience isolation and stigma, lacking social support and resources to navigate motherhood. The study tested the effectiveness of a community-based peer support intervention to mitigate social isolation and stigma of adolescent motherhood in Harare, Zimbabwe.

**Methods:**

Community health workers (n = 12) and peer educators (n = 12) in the intervention arm were recruited and trained on co-facilitating peer support groups. Adolescent mothers aged 15–18 years from two low-income high-density communities in Harare were recruited, using a quasi-experimental design. The intervention arm (n = 104) participated in the peer support groups and both arms completed sociodemographic, base-, mid-, and end-line surveys (control arm n = 79). Peer support groups (12 groups with 6–12 participants in each) met in-person twice a month and completed 12 sessions from May to August 2019 addressing participant-identified topics such as income generation and depression. WhatsApp Messenger was used for training and implementation support. Key community stakeholders discussed project progress and recommendations to improve adolescent mothers’ health. Data were analyzed using Stata 15.

**Results:**

The intervention arm reported lower depressive symptoms and common mental disorders and higher overall, family, friends, and significant-other support, compared to control. The intervention arm felt more engaged with peers, knew who and where to turn to for help, and had coping, parenting and communication strategies to manage life challenges.

**Conclusions for Practice:**

The intervention mitigated social isolation and stigma and thereby improved mental health and social support among adolescent mothers in Harare.

*Trial Registration* This trial is registered at Clinical Trials.gov, NCT05213182 https://clinicaltrials.gov/ct2/show/NCT05213182.

**Supplementary Information:**

The online version contains supplementary material available at 10.1007/s10995-023-03821-2.

## Significance

*What is already known on this subject*? Social isolation and stigma contribute to poor mental health outcomes. Adolescent mothers often experience isolation and stigma, lacking social support and resources to navigate motherhood. There is a need for interventions that identify and address the unique needs of adolescent mothers.

*What this study adds*? A community-based peer-support intervention can mitigate social isolation and stigma and thereby improve mental health and social support of adolescent mothers. WhatsApp Messenger is a potential intervention tool for providing training and implementation support, and enhancing communication between peer support group facilitators and participants.

**Supplementary Information:**

The online version contains supplementary material available at 10.1007/s10995-023-03821-2.

## Introduction

Approximately 12 million girls aged 15–19 years and at least 777,000 girls under 15 years give birth each year in low- and middle-income countries (WHO, 2020). The global adolescent fertility rate has declined by 11.6% over the past 20 years, but the number of child births to adolescents has not declined due to a growing adolescent population (WHO, 2020). The highest rates of early childbearing are found in sub-Saharan African countries (WHO, 2020). In Zimbabwe, almost a quarter of adolescent girls aged 15–19 years have begun childbearing, and these pregnancies are often unintended due to sexual abuse, risky sexual behavior, and early marriage (MHCC, 2016; ZIMSTAT, 2015).

Adolescent mothers in Zimbabwe often experience feelings of isolation because of forced marriage which is culturally intended to rectify the out-of-wedlock pregnancy. Almost a quarter of adolescent girls in Zimbabwe are married and these marriages result in the adolescent girl leaving her accustomed familial ties (MHCC, 2016). Adolescent mothers in Zimbabwe experience stigma from family, peers, community members and institutions such as schools and health facilities (Tinago et al., 2021). Similar experiences of stigma are documented among adolescent mothers globally (Cluver et al., 2008; Kola et al., 2020; Wiemann et al., 2005; Yardley, 2008). The stigma of adolescent pregnancy and motherhood in Zimbabwe leads to adolescent mothers lacking social support with a loss of social networks with 57.7% forced to leave their homes, 19.4% dropping out of school and 4.9% abandoned by their friends (MHCC, 2016; Tinago et al., 2021; ZIMSTAT, 2015). Adolescent mothers often lack coping skills and resources to successfully navigate motherhood. Unless addressed, these forces may have negative consequences for the mental health of the adolescent mother and downstream consequences for their children (Letourneau et al., 2004).

Social isolation and stigma contribute to physical, mental, and emotional health problems (Cluver et al., 2008; Hall-Lande et al., 2007; Kumar et al., 2018; Leigh-Hunt et al., 2017; Wiemann et al., 2005). Social isolation is linked to poorer mental health outcomes such as depressive symptoms and suicide attempts (Leigh-Hunt et al., 2017). The mental health infrastructure in Zimbabwe has limited human resources with only 12 psychiatrists and 16 psychologists, with a focus on treatment via a centralized mental health pathway where mental health treatment is mostly provided at central hospitals (Chibanda, 2017). Interventions are needed that identify and address the unique needs of adolescent mothers; engage with adolescent mothers to actively define their needs; develop their circle of care and connection (i.e., social support network); involve key community stakeholders such as parents and caregivers, education, religious and community leaders to address stigma related to mental illness and adolescent motherhood; and address mental human resource challenges and the prevention of mental illness.

In 2018, we conducted formative research utilizing focus groups with adolescent mothers, community health workers (CHWs), and key community stakeholders in a low-income, high-density community in Harare, Zimbabwe to understand the needs of adolescent mothers and how peer support groups could address those needs (Tinago et al., 2021). Participants identified potential support group topics that included income generation, mental health, sexual and reproductive health, and gossip. Preferred structure and methods for conducting the support groups included weekly or bi-weekly 1-h group sessions facilitated by a CHW and a peer educator at health and community centers. Use of WhatsApp Messenger to support intervention efforts was welcomed as an affordable, convenient, and user-friendly platform to share information if the messaging platform was private and not used to spread gossip, misinformation, and chain messages.

The aim of this study was to test the effectiveness of a community-based peer support intervention to mitigate social isolation and stigma of adolescent motherhood in Harare, Zimbabwe that was developed from the formative research. The hypothesis was that adolescent mothers who participate in the intervention will report feeling less lonely, more engaged with peers and significant caring adults in their lives, and more supported by peers and significant adults. They will also be more willing to ask for help, have reduced prevalence of depressive symptoms, and have new coping, parenting, and communication strategies to manage the challenges in their lives.

## Methods

### Setting

Zimbabwe is in south-east Africa and has a population of 12,973,808 (51.9% females) (ZIMSTAT, 2013). The study was conducted in two high-density, low-income, peri-urban communities in the capital city Harare. English is an official language of Zimbabwe and Shona is the main local language among Harare residents.

### Intervention Implementation

Evidence-based session plan curricula were developed by the research team in English and Shona to address the following 12 participant identified topics: (1) introduction to the peer support groups, (2) adolescent motherhood, (3) gossip, (4) healthy relationships, (5) depression, (6) substance abuse, (7) family planning, (8) sexual health, (9) healthy parenting, (10) income generation, (11) hygiene, and (12) moving forward as an adolescent mother (Online Resource 1). Subject matter experts were also sought to review and provide input on session plans. CHWs (n = 12) and peer educators (n = 12) were trained on implementing the curriculum by the project coordinator and local subject matter experts during a 3 day training session in February 2019. WhatsApp Messenger was used for facilitator training and implementation support, and as a platform for facilitators to communicate with each other to plan sessions. The project coordinator supported facilitators with additional monthly meetings to review session plans and project progress. Peer support groups (average 12 participants in each group) met in-person twice a month and completed 12 total 75-min peer-group sessions from May to August 2019. WhatsApp was used by participants and facilitators to schedule meetings, ask and respond to questions, and further discuss peer support group topics. Key community stakeholders met in May 2019 (two meetings) and August 2019 (two meetings) to discuss project progress and recommendations to improve adolescent mothers’ health in the intervention community. The Zimbabwe mental health referral pathway (Online Resource 1) was followed by ensuring that the individual was in a secure and supportive environment and referred to Parirenyatwa Hospital.

### Design

A quasi-experimental design was used to test differential changes over base-, mid-, and end-line in mental health and social support outcomes among adolescent mothers (14–18 years) in the intervention and control arms. A non-randomized unmasked parallel strategy was used to assign participants to the intervention and control arms. From the formative research that was previously conducted by the authors in the intervention community, community members identified adolescent pregnancy and mental health as key community issues (Tinago et al., 2021). The control community was similarly low-income and high-density in Harare and was located about 35 km from the intervention community.

### Sample

The sample was adolescent mothers aged 15–18 years (n = 104 intervention arm; n = 79 control arm) who were pregnant and/or had a child or children who resided in the two low-income high-density communities in Harare. CHWs (n = 12), and peer educators (n = 12) co-facilitated the peer support groups. CHWs had at least a 7th grade education and basic health training. Peer educators had given birth during adolescence, were between 19 and 25 years of age, with at least a 7th grade education. Key community stakeholders (n = 50) met during the intervention period to discuss project progress and recommendations to improve adolescent mothers’ health in the intervention community.

### Recruitment

The purposive sample was recruited in various locations such as pre- and postnatal clinics, and churches within the study communities (Online Resource 1). CHWs were recruited through a letter request to the City of Harare and interest meetings held at the local clinics in January 2019. Peer educators were recruited by the project coordinator and the CHWs in January 2019 through snowball sampling and in-person recruitment. Adolescent mothers were recruited by CHWs and peer educators, and through fliers, snowball sampling, and in-person recruitment between January and February 2019. Key community stakeholders were recruited by the CHWs and project coordinator in March and May 2019 via snowball sampling. Participants were screened for eligibility, and prior to participating, provided written consent; additional parental/guardian consent was provided for participants aged 14–17 years. The control arm received a US$5 gift card after completing each survey. Peer group participants received a US$10 personal care gift basket after completing all the sessions and transport reimbursement (approximately US$1) after each session. This study was registered at ClinicalTrials.gov (NCT05213182).

### Data Collection

Process evaluation measures such as facilitator session reports and research team member observations of the support group meetings tracked attrition and fidelity throughout this stage (Online Resource 1). Semi-structured interviews were also conducted with participants and facilitators in August and September 2019 for the process evaluation.

Surveys were developed by adapting existing, proven instruments to measure and test differential changes over baseline (March 2019), midline (May 2019), and endline (August 2019) in mental health and social support outcomes. The baseline and endline surveys were used for the analysis of intervention effects because 6 months of intervention was expected to be long enough to generate benefits; the midline survey was used for monitoring progress.

### Primary Outcomes

The primary outcomes were depressive symptoms and suicidal ideation, and perceived social support. Change from baseline to endline in depressive symptoms and suicide ideation was measured using the Patient Health Questionnaire (PHQ-9), a validated 9-item tool utilized to screen, diagnose, monitor, and measure depression severity and suicide ideation (Spitzer et al., 1999). The score of the PHQ-9 was the total of nine items each with responses 0–3, with higher scores meaning a worse outcome. Categories of the total score were: 0–4 minimal depression, 5–9 mild depression, 10–14 moderate depression, 15–19 moderately severe depression, and 20–27 severe depression. We defined a binary indicator of moderate-to-severe depression to be scores of 10–27 vs. 0–9. Change from baseline to endline in perceived social support was measured using the Multidimensional Scale of Perceived Social Support (MSPSS), a validated scale that measures perceived social support among participants’ friends, family, and significant others (Zimet et al., 1988). This scale had 12 items used to assess social support with a Likert scale. The scale measures social support from three categories: family, friends, and significant other. Scores from 1 to 2.9 were considered low support, 3 to 5 moderate support, and 5.1 to 7 high support, with higher scores meaning more support.

### Secondary Outcomes

The secondary outcomes were common mental disorders, and peer and significant adult support. Change from baseline to endline in common mental disorders was measured using the 14-item Shona Symptom Questionnaire (SSQ), a culturally appropriate common mental disorders screening tool (Patel et al., 1997). Participants scoring 7 or below were defined as not having a common mental disorder. Participants scoring 8 or more were defined as having a common mental disorder. Changes from baseline to endline in peer and significant adult support were measured using the Peer and Significant Adult Support (PSAS) Survey, a 12-item scale developed by the research team with a Likert scale of 0 (not at all), 1 (some of the time), 2 (most of the time), and 3 (all the time). The items include 1. I feel lonely, 2. I feel engaged with my peers, 3. I feel engaged with significant adults in my life, 4. I feel supported by my peers, 5. I feel supported by significant adults in my life, 6. I am willing to ask for help, 7. I know who I can turn to for help, 8. I know where I can turn to for help, 9. I have coping strategies to manage the challenges in my life, 10. I have parenting strategies to manage the challenges in my life, 11. I have communication strategies to manage the challenges in my life, and 12. I feel happy.

### Data Analysis

Socio-demographic and survey data were analyzed with Stata 15 between March and October 2019. Descriptive statistics were obtained as means or percentages. The differences between arms in the changes in outcomes from baseline to endline visits were analyzed using mixed-effects models with individual as the random effect and arm, visit, and arm times visit as fixed effects. The models for all outcomes were linear except for moderate-to-severe depression which was logistic. The arm-times-visit interaction estimated the difference between arms in the changes between baseline and endline (Gertler et al., 2016).

### Ethical Approval Mechanism

Approvals were sought and received from the West Chester University of Pennsylvania institutional review board, City of Harare ethics committee, University of Zimbabwe Ethics Committee, and the Medical Research Council of Zimbabwe prior to conducting research activities.

## Results

### Baseline Sociodemographic Characteristics of Facilitators, Intervention, and Control Participants

All the *facilitators* (n = 24), 12 each for CHWs and peer educators were female, from the intervention community, and of Shona ethnicity (Table 1). CHWs had a mean age of 50.5 years (range 27–72 years) and 63.63% were employed. Peer educators had a mean age of 21.75 years (range 19–24 years) and were unemployed (100.0%). Most CHWs were widowed (50.0%) and most peer educators were divorced or separated (58.3%). Most CHWs had a grade 7 education (50.0%), while most peer educators had received a high school education between Form 1–4 (91.7%).

**Table 1:** Socio-demographic characteristics of peer support group facilitators (N = 24)

Characteristic	Peer educators (n = 12)	Community health workers (n = 12)
	n (%)	n (%)
*Gender*		
Female	12 (100.0)	12 (100.0)
*Age*		
Mean age (years)	21.75	50.5
Age range (years)	19–24	27–72
*Race*		
Black	11 (91.7)	12 (100.0)
Multiracial	1 (8.3)	0
*Ethnicity*		
Shona	12 (100.0)	12 (100.0)
*Residency*		
Intervention community	12 (100.0)	12 (100.0)
*Marital status*		
Divorced or separated	7 (58.3)	1 (8.3)
Married—monogamous	3 (25.00)	5 (41.7)
Never married	2 (16.7)	0
Widowed	0	6 (50.0)
*Partner’s age*		
Mean age (years)	26	37.8
Age range (years)	24–28	30–47
*Highest education level*		
Primary (grade 1–7)	0	4 (33.3)
High school (form 1–4)	10 (83.3)	5 (41.7)
High school (form 5–6)	1 (8.3)	0
Bachelor’s	1 (8.3)	0
Other	0	3 (25.0)
*Religious affiliation*		
Catholic	5 (41.7)	3 (25.0)
Protestant	1 (8.3)	1 (8.3)
Pentecostal	6 (50.0)	4 (33.3)
Other	0	2 (16.7)
Missing	0	2 (16.7)
*Current employment*		
Yes	0	8 (66.7)
No	12 (100.0)	4 (33.3)
*Position*		
Physician	–	–
Registered nurse (RGN)	–	–
Midwife	–	1 (14.3)
Teacher	–	–
Other	–	6 (85.7)
*Length of held position*		
Average (years)	–	27.0
Range (years)	–	9–30
*Work with young women*		
Yes	1 (8.3)	9 (75.0)
No	11 (91.7)	2 (16.7)
Missing	0	1 (8.3)
*Have female child*		
Yes	0	4 (33.3)
No	12 (100.0)	7 (58.3)
Missing	0	1 (8.3)

The mothers in the intervention and control arms were similar at baseline. In the *intervention arm* (n = 142), the mean age was 17.6 years (range 15–18 years) (Table 2). Most were of Shona ethnicity (97.2%), 39.4% were in married monogamous relationships, and 91.6% had at least some high school education between Form 1–4. Most were not employed or earning an income (52.5%), had a previous pregnancy (91.5%), had other children (71.8%). 16.9% were pregnant at the time of screening. The mean months pregnant was 5.2. Most had a cell phone (69.0%) and 41.6% used WhatsApp. In the *control arm* (n = 105), the mean age was 17.5 years (range 15–18 years), most were of Shona ethnicity (87.6%), and most were in married monogamous relationships (63.8%). Most had at least some high school education between Form 1–4 (88.6%). 47.6% were unemployed with no source of income. 69.5% had a previous pregnancy. 35.2% were currently pregnant at the time of screening. The mean months pregnant was 6.1 months. Most had a cell phone (60.0%) and 44.8% used WhatsApp.

**Table 2:** Baseline sociodemographic characteristics of adolescent mothers (N = 247)

Characteristic	Intervention (n = 142)	Control (n = 105)
*Mean age (years)*	17.6	17.5
*Age range (years)*	15–18	15–18
*Race: Black*	100% (142)	100% (105)
*Ethnicity*		
Shona	97.2% (138)	87.6% (92)
Ndebele	1.4% (2)	3.8% (4)
Other	1.4% (2)	8.6% (9)
*Marital status*		
Never married	27.5% (39)	23.8% (25)
Married monogamous	39.4% (56)	63.8% (67)
Married polygamous	7.0% (10)	2.9% (3)
Divorced	25.4% (36)	9.5% (10)
Widowed	0.7% (1)	–
*Education*		
Primary (grade 1–7)	7.8% (11)	9.5% (10)
High school (form 1–4)	91.6% (130)	88.6% (93)
High school (form 5–6)	0.7% (1)	1.9% (2)
*Religion*		
Apostolic	17.6% (25)	15.2% (16)
Catholic	13.4% (19)	14.3% (15)
Protestant	10.6% (15)	21.0% (22)
Pentecostal	55.6% (79)	44.8% (47)
Traditional	2.1% (3)	1.9% (2)
Muslim	0.7% (1)	2.9% (3)
*Income source*		
None	53.5% (76)	47.6% (50)
Formally employed	1.4% (2)	1.9% (2)
Self-employed	21.8% (31)	17.1% (18)
Dependent	–	1.0% (1)
Other	20.4% (29)	32.4% (34)
Casual labor	2.1% (3)	–
Missing	0.7% (1)	–
*Had a previous pregnancy*	91.6% (130)	69.5% (73)
*Mean number of pregnancies (SD)*	1.1 (0.2)	1.1 (0.3)
*Has other children*	71.8% (102)	63.8% (67)
*Mean number of other children (SD)*	1.0 (0.2)	1.0 (0.2)
*Year last child was born*		
2014	2.0% (2)	–
2015	5.9% (6)	1.5% (1)
2016	13.7% (14)	–
2017	38.25% (39)	14.9% (10)
2018	34.3% (35)	65.7% (44)
2019	4.9% (5)	17.9% (12)
Missing	1.0% (1)	–
*Location of last born’s birth*		
Home	2.9% (3)	3.0% (2)
Clinic	52.9% (54)	55.2% (37)
Hospital	44.1% (45)	41.8% (28)
*Currently pregnant*	16.9% (24)	35.2% (37)
*Family knows of current pregnancy*	87.5% (21)	91.9% (34)
*Mean months pregnant*	5.2 (2.2)	6.1 (1.9)
*Attended an ANC visit*	45.8% (11)	91.9% (34)
*Mean month pregnant when attended first ANC visit*	5.8 (2.0)	5.3 (1.7)
*Average # of ANC visits*	1.4 (0.9)	1.8 (0.9)
*ANC frequency*		
Every 2 weeks	–	2.7% (1)
Every month	29.2% (7)	48.7% (18)
Other	16.7% (4)	40.5% (15)
N/A	54.2% (13)	2.7% (1)
Missing	–	5.4% (2)
*Has a cell phone*	69.0% (98)	60.0% (63)
*Uses WhatsApp*	41.6% (59)	44.8% (47)

### Differences in Changes from Baseline to Endline Surveys

In the *intervention arm*, 142, 110, and 104 participants completed the baseline, midline, and endline surveys, respectively (Table 3). In the *control arm*, 105, 88, and 79 participants completed the baseline, midline, and endline surveys, respectively. Participants’ involvement in determining format, frequency, and topics ensured that they were strongly and consistently engaged throughout the intervention, with high intervention arm cohort retention rates at midline (77%) and endline (73%). Cohort retention rates for the control arm were similar, 84% at midline and 73% at endline. The main reason for loss to follow up in both arms was participants moving outside the communities in search of economic activities.

**Table 3:** Baseline and endline estimates within arm and difference between arms in changes from baseline to endline (n = 183)

	Intervention (n = 104)		Control (n = 79)		Difference between arms in changes between baseline and endline	
	Baseline (SE)	End-line (SE)	Baseline (SE)	End-line (SE)	Estimate (SE)	p-value
*Depressive symptoms: PHQ-9 score (0–27)*	9.18 (0.450)	5.50 (0.490)	6.40 (0.446)	7.40 (0.603)	− 5.01 (0.905)	< 0.001
*Common mental disorders: SSQ score (0–14)*	8.10 (0.290)	6.38 (0.420)	6.31 (0.314)	7.43 (0.412)	− 3.10 (0.605)	< 0.001
*Perceived social support: MSPSS scores*						
Family (0–7)	4.86 (0.141)	5.43 (0.130)	5.41 (0.162)	4.90 (0.201)	1.03 (0.266)	< 0.001
Friends (0–7)	3.53 (0.171)	4.31 (0.155)	3.81 (0.234)	2.89 (0.203)	1.61 (0.352)	< 0.001
Significant other (0–7)	5.44 (0.118)	5.57 (0.115)	5.78 (0.136)	5.19 (0.205)	0.691 (0.229)	0.003
Overall (0–7)	4.61 (0.116)	5.10 (0.0974)	5.00 (0.134)	4.33 (0.169)	1.11 (0.218)	< 0.001
*Peer & significant adult support: PSAS scores*						
Q1 (0–3)	1.65 (0.0698)	2.09 (0.0750)	2.00 (0.0766)	2.05 (0.0944)	0.466 (0.137)	0.001
Q2 (0–3)	1.38 (0.0844)	1.86 (0.0790)	1.43 (0.0936)	1.48 (0.108)	0.388 (0.188)	0.041
Q3 (0–3)	1.63 (0.0773)	1.86 (0.0858)	2.01 (0.0903)	1.78 (0.119)	0.474 (0.169)	0.006
Q4 (0–3)	1.29 (0.0868)	1.63 (0.0888)	1.22 (0.104)	1.28 (0.125)	0.247 (0.202)	0.224
Q5 (0–3)	1.78 (0.0789)	2.00 (0.0831)	2.00 (0.0938)	1.91 (0.123)	0.310 (0.181)	0.089
Q6 (0–3)	1.92 (0.0662)	1.76 (0.0803)	1.71 (0.101)	1.72 (0.113)	− 0.173 (0.160)	0.279
Q7 (0–3)	1.32 (0.0792)	1.78 (0.0885)	1.72 (0.106)	1.21 (0.125)	0.907 (0.189)	< 0.001
Q8 (0–3)	1.21 (0.0839)	1.64 (0.0830)	1.63 (0.109)	1.09 (0.115)	0.917 (0.172)	< 0.001
Q9 (0–3)	1.02 (0.0813)	1.48 (0.0885)	1.07 (0.102)	0.901 (0.115)	0.644 (0.179)	< 0.001
Q10 (0–3)	1.03 (0.0848)	1.68 (0.0888)	1.04 (0.0956)	0.988 (0.120)	0.700 (0.177)	< 0.001
Q11 (0–3)	1.06 (0.0706)	1.61 (0.0813)	1.26 (0.0995)	1.05 (0.111)	0.798 (0.171)	< 0.001
Q12 (0–3)	1.41 (0.0728)	1.89 (0.0885)	1.70 (0.0846)	1.74 (0.111)	0.436 (0.149)	0.004
Overall (0–3)	1.39 (0.0462)	1.77 (0.0462)	1.57 (0.0540)	1.43 (0.0646)	0.508 (0.0884)	< 0.001

*Patient Health Questionnaire* (*PHQ-9*) Utilized to screen, diagnose, monitor, and measure the severity of depression. The score of the PHQ-9 was the total of nine items each with responses 0–3. Categories of the total score were: 0–4 minimal depression, 5–9 mild depression, 10–14 moderate depression, 15–19 moderately severe depression, 20–27 severe depression. Cronbach’s alpha of 0.839

*Shona Symptom Questionnaire* (*SSQ*) A culturally common mental disorders (CMD) screening tool that is intended for use in Zimbabwe. The SSQ questions are emic as well as etic, in which the 14-item SSQ are strong predictors of a mental health disorder. Participants who score 7 or below are defined as not having a CMD. Participants who score 8 or more are defined as having a CMD. The overall score was a mean. Cronbach’s alpha of 0.833

*Multidimensional Scale of Perceived Social Support* (*MSPSS*) A 12-item tool used to assess social support using a Likert scale. The tool measures social support from three categories: family, friends, and significant other. Results ranging from 1 to 2.9 could be considered low support; a score of 3–5 could be considered moderate support; a score from 5.1 to 7 could be considered high support. The subscores and overall score were means of items. Cronbach’s alpha of 0.890

*Peer and Significant Adult Support (PSAS) Survey* A self-report tool developed by the research team with adapted items from various scales and new items added to gauge participants’ peer and significant other support on a Likert scale of 0 (not at all), 1 (some of the time), 2 (most of the time), and 3 (all the time). Survey items include PSAS Q1-I feel lonely; PSAS Q2-I feel engaged with my peers; PSAS Q3-I feel engaged with significant adults in my life; PSAS Q4-I feel supported by my peers; PSAS Q5-I feel supported by significant adults in my life; PSAS Q6-I am willing to ask for help; PSAS Q7-I know who I can turn to for help; PSAS Q8-I know where I can turn to for help; PSAS Q9-I have coping strategies to manage the challenges in my life; PSAS Q10-I have parenting strategies to manage the challenges in my life; PSAS Q11-I have communication strategies to manage the challenges in my life; PSAS Q12-I feel happy. The overall score was the mean of the 12 items. Cronbach’s alpha of 0.817

The scores for the PHQ-9 and the SSQ had greater improvements (i.e., lower scores) in the intervention arm than the control arm by 5.01 (p < 0.001) and 3.10 (p < 0.001) points, respectively (Table 3). The probability of moderate-to-severe depression increased from baseline to endline in the control arm from 0.267 to 0.333 and decreased in the intervention arm from 0.408 to 0.231 (p = 0.005); as quantified by an odds ratio, the intervention participants were four times less likely to become moderately-to-severely depressed than were the control participants. Each of the four scores (i.e., family, friends, significant other, and total) for the MSPSS had greater improvements in the intervention arm than the control arm by 0.691 (p = 0.003), 1.03 (p < 0.001), 1.61 (p < 0.001), and 1.11 (p < 0.001) points, respectively. The overall score of the PSAS survey had greater improvement in the intervention arm than the control arm by 0.508 points (p < 0.001); 10 of the 12 sub-scores also had greater improvements in the intervention arm than in the control arm, i.e., all except feeling supported by peers (PSAS Q4) and willing to ask for help (PSAS Q6). Each scale used showed good reliability (all Cronbach’s alpha values were between 0.817 and 0.890).

### Participant Perspectives on the Peer Support Groups

Participants reported that the groups provided a new forum to address primary sources of stigma, and for sharing challenges and seeking advice, alleviating social isolation as a key source of stress. Participants named their peer support groups “Young Women of Today” to emphasize their identity as young women first, then mothers who face current issues. Participants and facilitators reported that the groups served as the sole source of sexual and reproductive health information for most participants. Key community stakeholders suggested that the intervention’s consistent community engagement and trust-building efforts led to increasingly positive and supportive attitudes towards the participants and the intervention concept. Facilitators reported that the detailed training and supervisory support provided (both in-person and via WhatsApp) were sufficient for purposes, and reported no significant challenges related to the frequency, location, or co-facilitation of the groups. Use of WhatsApp, which was welcomed considering its low cost and convenience, allowed continued conversations of session topics. All expressed a strong desire for the intervention to continue.

## Discussion

The community-based peer support intervention reduced social isolation and stigma of adolescent motherhood in the intervention arm. Mental health and social support among adolescent mothers in the intervention arm improved compared with the control arm. Similar to results from a study conducted in South Africa, participants successfully established a support network through peer group sessions which fostered the development of strong friendships (De La Rey & Parekh, 1996). Most studies globally have researched informational support from professionals on adolescent mother outcomes, and few researched peer support and its influence on adolescent mother’s mental health (Letourneau et al., 2004). Platforms for adolescent mothers in Zimbabwe to meet with peers, important supportive adults, and trained health workers to address topics that impact their mental health did not exist prior to this study. Existing adolescent mental health resources in Zimbabwe are specific to HIV and focus on treatment. Current resources include Lay Health Workers trained in providing and referring clients for mental health services through projects that include the Friendship Bench, Youth Friendship Bench, and Community Adolescent Treatment Supporters (Broström et al., 2021; Chibanda et al., 2015; Willis et al., 2019).

This study demonstrates the utility of WhatsApp Messenger as an intervention tool. Althought the coverage of WhatsApp for intervention arm participants was low at 41.6%, the use of WhatsApp was welcomed by most adolescent mothers and appreciated for its ease of communication and affordability. Additional research is needed to investigate how WhatsApp can be used effectively in intervention implementation in resource-poor settings and among vulnerable groups like adolescent mothers.

Community engagement through key community stakeholder involvement and support for the peer support groups contributed to the acceptance and success of the peer support group intervention. Adolescent mothers identified gaps in services to meet their needs which included income generation, skills building, and educational opportunities, parenting resources, and opportunities for them to meet with their peers to discuss concerns in their lives. Adolescent mothers welcomed the peer support groups as a platform to actively engage with their peers, receive education on various topics, and ask questions to knowledgeable and supportive adults.

This study was informed by formative research applying a community-based participatory method which engaged adolescent mothers to actively define their needs and develop strategies to address those needs, and involved CHWs and key community stakeholders to outline avenues to address adolescent pregnancy and improve adolescent mothers’s health (*insert citation here, blinded for review*). Participants’ involvement resulted in high cohort retention rates, which differs from other interventions which identify retention challenges when implementing programs for adolescent mothers (Asheer et al., 2014). This study used established and novel scales to measure primary and secondary outcomes, and addressed prevention of mental illness and health worker shortages that are common in resource-limited settings including Zimbabwe by training CHWs and peer educators on mental health and peer group facilitation.

Study limitations include the lack of recruitment of 14 year olds who were eligible for the study, but were challenging to recruit. This may be because they may not yet be open about their pregnancy status. Similar recruitment challenges are documented by Asheer et al. (2014) in two adolescent mother programs. About one quarter of participants were lost to follow up between baseline and endline in each arm, primarily to seek economic opportunities. The analytic method estimated intervention effects under the assumption that missing endline visits were at random. No important differences in outcome scores between participants who were and were not lost to follow up occurred at baseline in either arm (all p > 0.118, not shown). Therefore, loss to follow up likely did not bias estimation of intervention effects.

The peer support group intervention mitigated social isolation and stigma and thereby improved mental health and social support among adolescent mothers in Harare. All participants expressed a strong desire for the intervention to continue. Recommendations include expanding the intervention to similar low-income high-density communities in Zimbabwe, integration of these groups to existing services such as pre- and post-natal care, and adaptation of the peer-support group model to address the mental health needs for adolescents in general.

### Supplementary Information

Below is the link to the electronic supplementary material.

Supplementary file1 (PDF 80 KB)

## Data Availability

Not applicable.

## Abbreviations

ANC: Antenatal care
CHW: Community health worker
MSPSS: Multidimensional Scale of Perceived Social Support
PHQ-9: Patient Health Questionnaire
PSAS: Peer and Significant Adult Support Survey
SSQ: Shona Symptom Questionnaire
