# Supplementary material for: Testing the Effectiveness of a Community-Based Peer Support Intervention to Mitigate Social Isolation and Stigma of Adolescent Motherhood in Zimbabwe
Source: Matern Child Health J. 2023 Nov 13;28(4):657–66. doi: 10.1007/s10995-023-03821-2 (PMC10963463; doi:10.1007/s10995-023-03821-2)
Supplement: Supplementary file 1 — Supplementary file1 (PDF 80 KB) [file 10995_2023_3821_MOESM1_ESM.pdf]

Online Resource 1

Testing the Effectiveness of a Community-Based Peer Support Intervention to Mitigate Social Isolation and Stigma of Adolescent Motherhood in Zimbabwe

Maternal and Child Health Journal

Chiwoneso B. Tinago, PhD[1], Edward A. Frongillo, PhD[2], Andrea M. Warren, PhD[2], Vivian Chitiyo, MPhil[3], Tiara N. Jackson, PhD[4], Ashley K. Cifarelli, MPH[1], Shannon Fyalkowski, MPH[1], Victoria Pauline, MPH[1]

[1]Department of Public Health Sciences, West Chester University of Pennsylvania, West Chester, PA, USA
[2]Department of Health Promotion, Education, and Behavior, University of South Carolina, Arnold School of Public Health, Columbia, SC, USA
[3]The Organization for Public Health Interventions and Development (OPHID), Harare, Zimbabwe
[4]NORC at the University of Chicago, Bethesda, MD, USA

Chiwoneso B. Tinago, PhD (corresponding author)
Associate Professor
Department of Public Health Sciences
West Chester University of Pennsylvania
Address: 155 University Avenue
West Chester, PA 19383, United States
Phone: +1610-436-2776
Fax: +1610-436-2860
E-mail: ctinago@wcupa.edu

Intervention Details

The intervention curriculum was developed by the research team who adapted existing evidence-based curricula addressing each of the participant identified session topics. For example, the family planning session plan included information and activities adapted from the Advocates for Youth, Life Planning Education & Family Life and Sexual Health lesson plan and the session plan on hygiene was adapted from Save the Children: The School Health and Nutrition Health Education Manual. Subject matter experts were also sought to review and provide input on session plans. For example, a Zimbabwean child psychologist reviewed sessions plans on mental health. The Project Coordinator who was a member of the research team and based in Zimbabwe, provided technical support to the CHWs, peer educators and participants, including training facilitators on implementing the peer support groups and session plans, responding to questions about the project, and coordinating and monitoring WhatsApp communication.

Mental Health Referral Pathway for Zimbabwe

A total of 48 participants were referred to mental health services during the study period. The mental health referral pathway for Zimbabwe was followed for participants who were screened and were found

to have depression, common mental disorder or be suicidal. Contact information or the referral service were provided. This referral pathway included the following:

1. Ensure that the individual is in a secure and supportive environment.
2. Make sure the individual is not left alone.
3. Be empathetic and non-judgmental.
4. With the individual's permission, mobilize and involve close family and friends for social support.
5. Where possible, remove access to means of self- harm.
6. Refer for evaluation at the nearest mental health facility (Parirenyatwa Hospital – contact information provided).

For attempted suicide:

1. Ensure the individual's safety.
2. Ensure the individual is transported as quickly as possible to the nearest emergency health facility for medical stabilization (Parirenyatwa Hospital).

Recruitment Details

CHWs were recruited through a letter request to the City of Harare (since they are an official health cadre of the City of Harare Health Services) and interest meetings held at the local clinics in January 2019. Peer educators were recruited by the project coordinator and the CHWs in January 2019 through snowball sampling and in-person recruitment. Adolescent mothers were recruited by CHWs and peer educators, and through fliers, snowball sampling, and in-person recruitment through home visits, at pre- and postnatal clinics, and churches within the study communities between January and February 2019. Clinic staff would also share information about the project to women seeking pre- and post-natal care. Snowball sampling included peer-educators and adolescent mothers sharing project information (contact number of project coordinator or referring them to the local clinic for more information) with other adolescent mothers in the community. The control group received a US$5 gift card incentive after completing each survey. Peer support group participants received a personal care gift basket valued at US$10 after completing all the peer group sessions and transport reimbursement (approximately US$1) during each of their sessions.

Fidelity of Curriculum Delivery

Fidelity was assessed with observations of group sessions and facilitator session reports. Each group was observed at least once by a research team member using an observation guide. The observation reports highlighted that the session plans were conducted as described in the session plan. Five categories were assessed on a Likert scale of 1-3, with 1 being not observed and 3 being clearly observed: A) Design & Facilitation, B) Community Health Worker (CHW) Actions, C) Peer Educators (PE) Actions, D) Participants' Actions, E) Overall Assessment. Together, the aforementioned categories averaged 2.87 on a scale of 3. Facilitator session reports were conducted for all sessions and discussed with the project coordinator during the monthly facilitator meetings. No challenges were reported with implementing the peer groups as outlined in the session plans.
